# Association between steatotic liver disease subtype and thyroid cancer in women: A nationwide population-based cohort study

**DOI:** 10.3389/fendo.2026.1801839

**Published:** 2026-05-21

**Authors:** Hyunju Park, Arim Choi, Soo-Kyung Kim, Yong-Wook Cho, Kyungdo Han, Kyung-Soo Kim

**Affiliations:** 1 Department of Internal Medicine, CHA Bundang Medical Center, CHA University School of Medicine, Seongnam, Republic of Korea; 2 Department of Statistics and Actuarial Science, Soongsil University, Seoul, Republic of Korea

**Keywords:** alcoholic liver disease, fatty liver, nonalcoholic fatty liver disease, steatosis of liver, thyroid cancer

## Abstract

**Objective:**

This study aimed to investigate association between steatotic liver disease (SLD) subtype and thyroid cancer in women using a nationwide population-based cohort.

**Methods:**

This nationwide population-based cohort study included 2,089,609 women aged more than 40 years who participated in the 2009 health screening program and were followed up until 2022. Participants were divided into four groups based on their SLD status: no SLD, metabolic dysfunction-associated steatotic liver disease (MASLD), metabolic dysfunction and alcohol-related liver disease (MetALD), and alcohol-related liver disease (ALD).

**Results:**

During a mean follow-up of 12.27 years, 40,072 (1.92%) women were newly diagnosed with thyroid cancer. Compared with the no SLD group, women with MASLD had a higher risk of thyroid cancer (hazard ratio [HR]: 1.19, 95% confidence interval [CI]: 1.16-1.22), whereas those with MetALD and ALD did not have a higher risk. The risk of thyroid cancer was higher in women with MASLD, including both premenopausal (HR: 1.21, 95% CI: 1.16-1.26) and postmenopausal women (HR: 1.17, 95% CI: 1.14-1.21). Women in the MetALD group showed no risk elevation of thyroid cancer regardless of their menopausal status. Postmenopausal women in the ALD group had a higher risk of thyroid cancer than those in the no SLD group (HR: 1.31, 95% CI: 1.05-1.64), whereas premenopausal women did not have a higher risk.

**Conclusions:**

Thyroid cancer risk was modestly increased in women with MASLD, whereas no significant associations were observed for MetALD or ALD.

## Introduction

The prevalence of thyroid cancer has significantly increased over the last few decades (1–3). This is mainly attributed to earlier detection of small-sized thyroid cancer using high-resolution ultrasonography, combined with increased medical surveillance (4–6). However, prevalence rates have also increased for more advanced and larger tumors (7). Thus, overdiagnosis alone cannot fully explain the increase in prevalence. Other risk factors need to be considered.

Several potential risk factors for the increase in incidence of thyroid cancer have been reported, including genetic alterations, radiation exposure, iodine intake, and female sex (8). Furthermore, many studies have reported that higher body mass index (BMI) and metabolic syndrome are associated with an increased risk of thyroid cancer (9–11). Steatotic liver disease (SLD) is commonly associated with metabolic disorders driven by insulin resistance (12, 13). Based on this, a previous study has reported that nonalcoholic fatty liver disease (NAFLD), which is associated with insulin resistance and obesity, is a risk factor for thyroid cancer in individuals aged 20 to 39 years (14).

Recently, metabolic dysfunction-associated steatotic liver disease (MASLD) was introduced as a new nomenclature to provide a clearer classification of SLD (15). Metabolic dysfunction and alcohol-related liver disease (MetALD) and alcohol-related liver disease (ALD) have also been introduced to describe patients with SLD who consume excessive alcohol. Previous studies have assessed the risk of thyroid cancer in patients with NAFLD, which can reflect underlying metabolic dysfunction such as insulin resistance. In addition, several studies have reported an inverse association between alcohol consumption and the risk of thyroid cancer (16–18). However, no studies have evaluated this association using the newly defined SLD subtypes that account for both metabolic disorders and alcohol consumption. Therefore, this study aimed to investigate the association between SLD subtype and the risk of thyroid cancer in women using a nationwide population-based cohort.

## Materials and methods

### Data sources

This nationwide, population-based cohort study used data from the Korean National Health Information Database (NHID). The NHID is a database that integrates information from the National Health Insurance (NHIS) and National Health Screening Program. NHIS as part of the social security system is a single insurer in Korea that covers 97% of the population (19). The NHID includes sociodemographic variables, diagnoses coded according to the 10^th^ edition of the International Classification of Disease (ICD-10), and prescription records. Medical interviews, anthropometric measurements, blood tests, and additional assessments were included in the NHIS. Details on these variables have been published in previous studies (20–22). This study was approved by the Institutional Review Board (IRB) of CHA Bundang Medical Center (CHAMC 2025-03-058). The requirement of informed consent was waived by the IRB because all data were de-identified.

### Study design and population

A total of 3,109,491 women aged over 40 years who participated in the National Health Screening Program in 2009 were selected. Among them, those with missing data on menopausal status (n = 82,349), those with hysterectomy (n = 204,288), those with menarche < 5 years or ≥ 30 year and those with menopause < 30 years or > 60 years (n = 181,064), those with hepatocellular carcinoma (n = 1,219), those with liver transplantation (n = 76), and those with missing data (n = 417,384) were excluded. Women with concomitant liver disease within one year prior to screening (n = 83,932), women with no metabolic SLD (n = 706), and women with any cancer wash-out (n = 42,794) were also excluded. To overcome bias, 6,070 women diagnosed with thyroid cancer within one year after study enrollment were additionally excluded. Finally, a total of 2,089,609 women were included in this study (**Supplementary Figure 1**).

### Measurements

Standardized self-reported questionnaires were used to obtain information on smoking status, alcohol consumption, regular exercise, income, age at menarche, age at menopause, parity, duration of breastfeeding, duration of oral contraceptive use, and duration of hormone replacement therapy (HRT). An ex-smoker was defined as a participant who had quit smoking before health examination. Alcohol consumption was categorized into three groups: mild (< 20 g/day), moderate (20–50 g/day), and heavy (≥ 50 g/day) drinkers. Regular exercise was defined as engaging in moderate-intensity activity for more than 30 minutes at least five times per week, or high-intensity activity for more than 20 minutes at least three times per week. Low income was defined as the lowest 20% of the total population based on monthly income.

The National Health Screening Program included laboratory tests, such as fasting blood glucose, total cholesterol, triglycerides, high-density lipoprotein cholesterol, low-density lipoprotein cholesterol, aspartate aminotransferase, alanine aminotransferase, γ-glutamyl transferase, and creatinine levels. Estimated glomerular filtration rate was calculated using the Chronic Kidney Disease Epidemiology Collaboration equation.

### Definitions

Participants were divided into four groups: no SLD, MASLD, MetALD, and ALD. Hepatic steatosis wasdefined as a fatty liver index (FLI) ≥ 30. The FLI was calculated using the followingequation: (e^0.95×log e (TG)+0.139 × BMI + 0.718×log e (GGT) +0.053×WC−15.745^)/(1 + e^0.95×log e (TG)+0.139×BMI+0.718×log e (GGT) +0.053×WC − 15.745^) × 100 (23). Participants with FLI < 30 were classified as the no SLD group. MASLD was defined as the presence of hepatic steatosis with one or more of the following cardiometabolic risk factors: BMI ≥ 23 kg/m^2^ or waist circumference > 85 cm for females (24); fasting glucose ≥ 100 mg/dL, diagnosis with diabetes, or use of glucose-lowering drugs; blood pressure ≥ 130/85 mmHg or use of antihypertensive drugs; triglyceride ≥ 150 mg/dL; or high-density lipoprotein cholesterol ≤ 50 mg/dL for females or use of lipid-lowering drugs. Those with greater amounts of alcohol intake (weekly intake ≥ 140 g for females) were excluded from the MASLD group. Participants with SLD and one or more cardiometabolic risk factors who reported greater amounts of alcohol intake (weekly intake 140–350 g) were classified as MetALD. Participants with SLD and one or more cardiometabolic risk factors who reported excessive alcohol intake (weekly intake ≥ 350 g) or were diagnosed with alcohol abuse, misuse, or alcohol-related liver disease were classified as ALD.

BMI was calculated as body weight (kg) divided by height (m) squared. Obesity was defined as BMI ≥ 25 kg/m^2^. Diabetes mellitus was defined as a fasting plasma glucose concentration ≥ 126 mg/dL or the presence of at least one prescription claim per year for antidiabetic drugs under ICD-10 codes E11-14. Hypertension was defined as blood pressure ≥ 140/90 mm Hg or the presence of at least one prescription claim per year for antihypertensive drugs under ICD-10 codes I10–13 or I15. Dyslipidemia was defined using ICD-10 code E78 and at least one prescription claim per year for a lipid-lowering agent or a total cholesterol level ≥ 240 mg/dL.

### Study outcome

The primary outcome was incident thyroid cancer. Thyroid cancer was defined as the presence of both the ICD-10 code C73 and the cancer-specific insurance claim code V193. However, information on the stage of thyroid cancer was not available. Its incidence was evaluated based on claim records from the NHIS during the follow-up period. Participants were followed from baseline to the date of thyroid cancer diagnosis or until December 31, 2022, whichever came first.

### Statistical analysis

Continuous variables are presented as mean ± standard deviation or geometric mean (95% confidential interval [CI]). Categorical variables are presented as numbers with percentages. An Analysis of Variance (ANOVA) was performed to compare continuous variables, while categorical variables were compared using the chi-square test. Incidence rates are presented as number of events occurring per 1,000 person-years. Multivariable Cox proportional hazard regression models were used to evaluate hazard ratio (HR) and 95% CI for the incidence of thyroid cancer. Model 1 was unadjusted. Model 2 was adjusted for age. Model 3 was adjusted for age, income, smoking status, regular exercise, parity, duration of breastfeeding, duration of oral contraceptive use, and age at menarche in total and premenopausal participants. In postmenopausal participants, age at menopause and duration of HRT were additionally adjusted in Model 3. Kaplan-Meier survival curves were constructed to compare cumulative incidence rates of thyroid cancer according to SLD subtype. Subgroup analysis was performed based on income, smoking status, regular exercise, obesity, parity, duration of breastfeeding, duration of oral contraceptive use, and duration of HRT. Statistical significance was considered when a two-sided *p*-value was less than 0.05. All statistical analyses were performed using SAS version 9.4 (SAS Institute, Cary, NC, USA).

## Results

Baseline characteristics of the study population are summarized in **Table 1**. The mean age of the study population was 54.8 years and the mean BMI was 23.8 kg/m^2^. Among them, 76.4% (n = 1,597,364) had no SLD, 22.8% (n = 476,874) had MASLD, 0.4% (n = 9,017) had MetALD, and 0.3% (n = 6,354) had ALD. Women with SLD were older with higher BMI and higher proportions of obesity, diabetes mellitus, hypertension, and dyslipidemia than those with no SLD (**Supplementary Table 1**). Metabolic parameters, including blood pressure, glucose level, and lipid profiles were worse in women with SLD than in those without SLD. Age, BMI, and waist circumference were higher in patients with MASLD than in those without SLD. Metabolic diseases including obesity, diabetes mellitus, hypertension, and dyslipidemia were also more common in patients with MASLD than in those without SLD. Age of the MetALD group was similar to that of the group without SLD. However, BMI and waist circumference were higher in the MetALD group than in the group without SLD. Metabolic diseases including obesity, diabetes mellitus, hypertension, and dyslipidemia were also more common in the MetALD group than in the group without SLD. However, metabolic diseases tended to be less common in women with MetALD than in those with MASLD. In women with ALD, their clinical characteristics were similar to those of women with MetALD, although women with ALD tended to be slightly older than those with MetALD.

**Table 1:** Baseline characteristics according to steatotic liver disease subtype.

Characteristics	Total (n=2,089,609)	No SLD (n=1,597,364)	MASLD (n=476,874)	MetALD (n=9,017)	ALD (n= 6,354)	P value
Age, years	54.8 ± 10.8	53.5 ± 10.6	59.2 ± 10.3	51.4 ± 8.3	56.2 ± 9.9	< 0.001
BMI, kg/m^2^	23.8 ± 3.2	22.7 ± 2.4	27.2 ± 2.9	26.6 ± 3.2	26.6 ± 3.2	< 0.001
Waist circumference, cm	78.0 ± 8.4	75.2 ± 6.5	87.4 ± 6.9	85.8 ± 7.5	86.3 ± 7.6	< 0.001
Smoking status						< 0.001
Never	2,001,499 (95.8)	1,533,826 (96.0)	456,284 (95.7)	6,286 (69.7)	5,103 (80.3)	
Former	26,449 (1.3)	19,911 (1.3)	5,865 (1.2)	484 (5.4)	189 (3.0)	
Current	61,661 (3.0)	43,627 (2.7)	14,725 (3.1)	2,247 (24.9)	1,062 (16.7)	
Alcohol consumption						< 0.001
None	1,692,185 (81.0)	1,282,538 (80.3)	406,485 (85.2)	0 (0.0)	3,162 (49.8)	
Mild	362,939 (17.4)	291,521 (18.3)	70,389 (14.8)	0 (0.0)	1,029 (16.2)	
Moderate	29,801 (1.4)	20,414 (1.3)	0 (0.0)	9,017 (100.0)	370 (5.8)	
Heavy	4,684 (0.2)	2,891 (0.2)	0 (0.0)	0(0.0)	1,793 (28.2)	
Regular exercise	372,125 (17.8)	293,440 (18.4)	75,983 (15.9)	1,665 (18.5)	1,037 (16.3)	< 0.001
Low income	392,919 (18.8)	304,605 (19.1)	85,142 (17.9)	1,874 (20.8)	1,298 (20.4)	< 0.001
Obesity	668,290 (32.0)	281,264 (17.6)	376,272 (78.9)	6,301 (69.9)	4,453 (70.1)	< 0.001
Diabetes mellitus	186,244 (8.9)	92,780 (5.8)	90,836 (19.1)	1,200 (13.3)	1,428 (22.5)	< 0.001
Hypertension	677,549 (32.4)	405,289 (25.4)	264,388 (55.4)	4,174 (46.3)	3,698 (58.2)	< 0.001
Dyslipidemia	490,815 (23.5)	295,850 (18.5)	189,293 (39.7)	2,791 (31.0)	2,881 (45.3)	< 0.001
SBP, mmHg	122.2 ± 16.0	120.1 ± 15.4	129.2 ± 16.0	128.8 ± 16.2	128.5 ± 16.0	< 0.001
DBP, mmHg	75.4 ± 10.3	74.2 ± 10.0	79.2 ± 10.1	80.5 ± 10.7	79.6 ± 10.4	< 0.001
Fasting glucose, mg/dL	97.2 ± 22.1	94.7 ± 18.7	105.2 ± 29.2	105.5 ± 28.1	107.8 ± 30.1	< 0.001
Total cholesterol, mg/dL	201.6 ± 37.7	197.8 ± 36.0	213.8 ± 40.4	213.0 ± 39.6	210.2 ± 42.4	< 0.001
Triglyceride, mg/dL	104.1 (104.1-104.2)	90.0 (89.9-90.1)	167.3 (167.1-167.5)	166.9 (165.2-168.7)	169.3 (167.2-171.3)	< 0.001
HDL cholesterol, mg/dL	58.6 ± 32.1	59.7 ± 29.8	54.7 ± 38.3	61.5 ± 35.9	58.0 ± 35.9	< 0.001
LDL cholesterol, mg/dL	120.5 ± 37.8	119.4 ± 36.3	124.5 ± 42.0	115.1 ± 39.7	115.9 ± 44.3	< 0.001
eGFR, mL/min/1.73m2	84.9 ± 28.8	85.7 ± 28.6	82.0 ± 28.9	87.7 ± 29.4	83.7 ± 27.8	< 0.001
AST, IU/L	22.3 (22.3-22.3)	21.5 (21.4-21.5)	25.3 (25.3-25.4)	26.8 (26.6-27.1)	29.6 (29.2-30.0)	< 0.001
ALT, IU/L	18.6 (18.5-18.6)	17.0 (17.0-17.0)	24.6 (24.6-24.7)	24.9 (24.6-25.1)	27.9 (27.5-28.3)	< 0.001
r-GTP, IU/L	19.1 (19.1-19.1)	16.6 (16.6-16.6)	30.0 (29.9-30.0)	48.1 (47.4-48.9)	49.1 (48.1-50.2)	< 0.001
Age at menarche, years	15.9 ± 1.9	15.8 ± 1.9	16.2 ± 1.9	15.9 ± 1.9	16.2 ± 1.9	< 0.001
Menopause	1,220,141 (58.4)	845,856 (53.0)	365,627 (76.7)	4,346 (48.2)	4,312 (67.9)	< 0.001
Parity						< 0.001
No	51,866 (2.5)	43,099 (2.7)	8,184 (1.7)	351 (3.9)	232 (3.7)	
1	187,123 (9.0)	154,376 (9.7)	30,838 (6.5)	1,257 (13.9)	652 (10.3)	
≥ 2	1,850,620 (88.6)	1,399,889 (87.6)	437,852 (91.8)	7,409 (82.2)	5,470 (86.1)	
Breastfeeding, months						< 0.001
0	232,798 (11.1)	193,473 (12.1)	37,143 (7.8)	1,394 (15.5)	788 (12.4)	
< 6	289,595 (13.9)	251,091 (15.7)	36,813 (7.7)	1,160 (12.9)	531 (8.4)	
6 – 12	436,010 (20.9)	353,369 (22.1)	79,910 (16.8)	1,700 (18.9)	1,031 (16.2)	
≥ 12	1,131,206 (54.1)	799,431 (50.1)	323,008 (67.7)	4,763 (52.8)	4,004 (63.0)	
Oral contraceptive use, months						< 0.001
0	1,789,179 (85.6)	1,375,807 (86.1)	401,343 (84.2)	6,918 (76.7)	5,111 (80.4)	
< 12	195,858 (9.4)	148,443 (9.3)	45,539 (9.6)	1,173 (13.0)	703 (11.1)	
≥ 12	104,572 (5.0)	73,114 (4.6)	29,992 (6.3)	926 (10.3)	540 (8.5)	

Continuous variables are expressed as mean ± standard deviation or geometric mean (95% confidential interval). Categorical data are presented as frequencies and percentages.

BMI, body mass index; SBP, systolic blood pressure; DBP, diastolic blood pressure; HDL, high-density lipoprotein; LDL, low-density lipoprotein; eGFR, estimated glomerular filtration rate; AST, aspartate aminotransferase; ALT, alanine aminotransferase; r-GTP, gamma-glutamyl transferase; SLD, steatotic liver disease; MASLD, metabolic dysfunction-associated steatotic liver disease; MetALD, metabolic alcohol-associated liver disease; ALD, alcohol-related liver disease.

**Figure 1:**
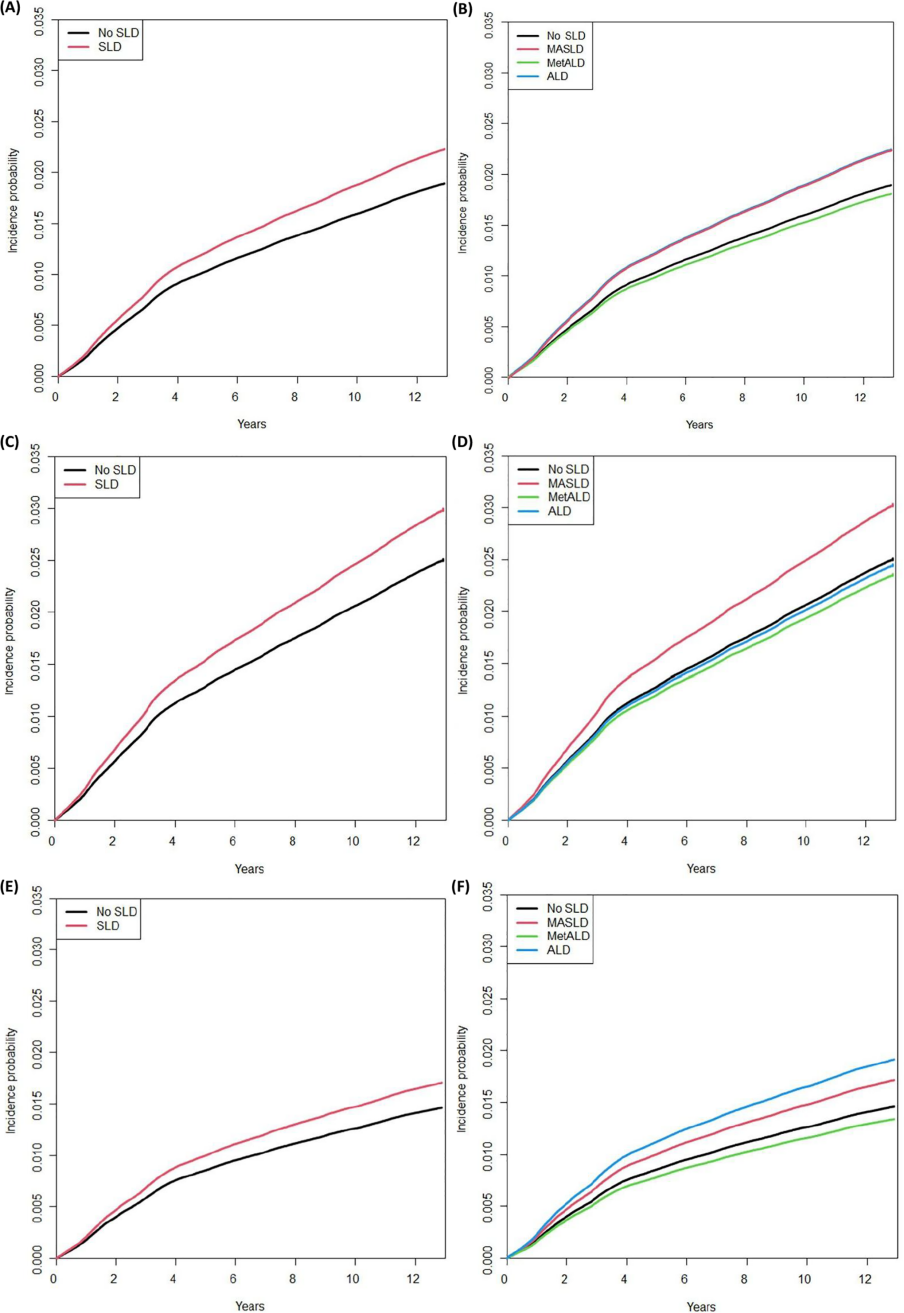
Cumulative incidence of thyroid cancer according to the presence of steatotic liver disease (SLD) and subtype in total **(A, B)**, premenopausal women **(C,D)**, and postmenopausal women (E,F). **(A)** total: no SLD vs. SLD (P<0.001); **(B)** total: no SLD vs. MASLD vs. MetALD vs. ALD (P<0.001); **(C)** premenopausal women: no SLD vs. SLD (P<0.001); **(D)** premenopausal women: no SLD vs. MASLD vs. MetALD vs. ALD (P<0.001); **(E)** postmenopausal women: no SLD vs. SLD (P<0.001); **(F)** postmenopausal women: no SLD vs. MASLD vs. MetALD vs. ALD (P<0.001). MASLD, metabolic dysfunction-associated steatotic liver disease; MetALD, metabolic alcohol-associated liver disease; ALD, alcohol-related liver disease.

During a mean follow-up of 12.27 years, 40,072 (1.9%) women were newly diagnosed with thyroid cancer (**Table 2**). The cumulative incidence of thyroid cancer was higher in women with SLD than in those without SLD (*P* < 0.001) (**Figure 1A**). The unadjusted HR for thyroid cancer in women with SLD was 0.97 (95% CI: 0.95-0.99). However, after adjustment for age, the HR for thyroid cancer in women with SLD was 1.18 (95% CI: 1.15-1.21) (Model 2). In the multivariable adjusted model (Model 3), women with SLD still showed an elevated HR for thyroid cancer (HR: 1.18, 95% CI: 1.15-1.21). The incidence rate of thyroid cancer per 1,000 person-years was 1.57 in women with no SLD, 1.53 in those with MASLD, 1.49 in those with MetALD, and 1.61 in those with ALD. The cumulative incidence of thyroid cancer in women with MASLD was higher than that in those without SLD (*P* < 0.001) (**Figure 1B**). After adjusting for age, income, smoking status, regular exercise, parity, duration of breastfeeding, duration of oral contraceptive use, and age at menarche, women with MASLD (HR: 1.19, 95% CI: 1.16-1.22) were associated with a greater risk of thyroid cancer, whereas women with MetALD and ALD were not.

**Table 2:** Risk of thyroid cancer according to the presence of steatotic liver disease and subtype.

	Number	Event	Duration (person-years)	Incidence rate^*^	Hazard ratio (95% Confidence interval)		
					Model 1	Model 2	Model 3
No SLD	1,597,364	30,831	19,579,424.8	1.57	1 (Reference)	1 (Reference)	1 (Reference)
SLD	492,245	9,241	6,055,503.8	1.53	0.97 (0.95-0.99)	1.18 (1.15-1.21)	1.18 (1.15-1.21)
SLD subtype							
No SLD	1,597,364	30,831	19,579,424.8	1.57	1 (Reference)	1 (Reference)	1 (Reference)
MASLD	476,874	8,950	5,866,719.8	1.53	0.97 (0.95-0.99)	1.19 (1.16-1.22)	1.19 (1.16-1.22)
MetALD	9,017	165	110,618.6	1.49	0.95 (0.81-1.11)	0.90 (0.77-1.05)	0.96 (0.82-1.11)
ALD	6,354	126	78,165.4	1.61	1.03 (0.86-1.22)	1.14 (0.95-1.35)	1.19 (1.00-1.42)

Model 1: no adjustment;.

Model 2: adjusted for age;

Model 3: adjusted for age, income, smoking status, regular exercise, parity, duration of breastfeeding, duration of oral contraceptive use, and age at menarche.

SLD, steatotic liver disease; MASLD, metabolic dysfunction-associated steatotic liver disease; MetALD, metabolic alcohol-associated liver disease; ALD, alcohol-related liver disease.

^*^ Incidence per 1000 person years.

Participants were further divided into two groups based on menopausal status (**Supplementary Table 2**). Of 2,089,609 participants, 58.4% (n = 1,220,141) were postmenopausal women. Among premenopausal women, 86.4% had no SLD, 12.8% had MASLD, 0.5% had MetALD, and 0.2% had ALD. In postmenopausal women, 69.3% had no SLD, 30.0% had MASLD, 0.4% had MetALD, and 0.4% had ALD. Obesity, diabetes mellitus, hypertension, and dyslipidemia were more common in postmenopausal women than in premenopausal women. The cumulative incidence of thyroid cancer in both premenopausal and postmenopausal women with SLD was higher than that in those without SLD (*P* < 0.001) (**Figures 1C, E**). The risk of thyroid cancer was higher in both premenopausal women (HR: 1.21, 95% CI: 1.16-1.26) and postmenopausal women (HR: 1.17, 95% CI: 1.14-1.21) with MASLD (**Table 3**). However, MetALD did not elevate the risk of thyroid cancer in either premenopausal women or postmenopausal women. In the ALD group, the risk of thyroid cancer was higher in postmenopausal women (HR: 1.31, 95% CI: 1.05-1.64), whereas no increased risk was observed in premenopausal women. Kaplan-Meier survival curves showed that MASLD was associated with a higher risk of thyroid cancer than no SLD in both premenopausal and postmenopausal women (all *P* < 0.001) (**Figures 1D, F**). Postmenopausal women with ALD had a higher risk of thyroid cancer, whereas premenopausal women with ALD did not.

**Table 3:** Risk of thyroid cancer according to the presence of steatotic liver disease and subtype in premenopausal and postmenopausal women.

	Number	Event	Duration (person-years)	Incidence rate^*^	Hazard ratio (95% Confidence interval)		
					Model 1	Model 2	Model 3
Premenopausal women							
No SLD	751,508	18,267	9,143,432.2	2.00	1 (Reference)	1 (Reference)	1 (Reference)
SLD	117,960	3,417	1,431,878.0	2.39	1.19 (1.15-1.24)	1.19 (1.15-1.23)	1.20 (1.15-1.24)
SLD subtype							
No SLD	751,508	18,267	9,143,432.2	2.00	1 (Reference)	1 (Reference)	1 (Reference)
MASLD	111,247	3,270	1,349,851.6	2.42	1.21 (1.17-1.26)	1.21 (1.16-1.25)	1.21 (1.16-1.26)
MetALD	4,671	101	57,055.2	1.77	0.89 (0.73-1.08)	0.89 (0.73-1.08)	0.94 (0.77-1.14)
ALD	2,042	46	24,971.2	1.84	0.92 (0.69-1.23)	0.92 (0.69-1.23)	0.98 (0.73-1.30)
Postmenopausal women							
No SLD	845,856	12,564	10,435,992.6	1.20	1 (Reference)	1 (Reference)	1 (Reference)
SLD	374,285	5,824	4,623,625.8	1.26	1.05 (1.02-1.08)	1.17 (1.13-1.20)	1.17 (1.13-1.21)
SLD subtype							
No SLD	845,856	12,564	10,435,992.6	1.20	1 (Reference)	1 (Reference)	1 (Reference)
MASLD	365,627	5,680	4,516,868.2	1.26	1.05 (1.01-1.08)	1.17 (1.13-1.21)	1.17 (1.14-1.21)
MetALD	4,346	64	53,563.4	1.19	0.99 (0.78-1.27)	0.85 (0.67-1.10)	0.92 (0.72-1.17)
ALD	4,312	80	53,194.2	1.50	1.25 (1.00-1.56)	1.26 (1.01-1.57)	1.31 (1.05-1.64)

Model 1: no adjustment;.

Model 2: adjusted for age;

Model 3 adjusted for age, income, smoking status, regular exercise, parity, duration of breastfeeding, duration of oral contraceptive use, and age at menarche in premenopausal women; adjusted for age, income, smoking status, regular exercise, parity, duration of breastfeeding, duration of oral contraceptive use, age at menarche, age at menopause, and hormone replacement therapy in postmenopausal women.

SLD, steatotic liver disease; MASLD, metabolic dysfunction-associated steatotic liver disease; MetALD, metabolic alcohol-associated liver disease; ALD, alcohol-related liver disease.

^*^ Incidence per 1000 person years

**Figure 2:**
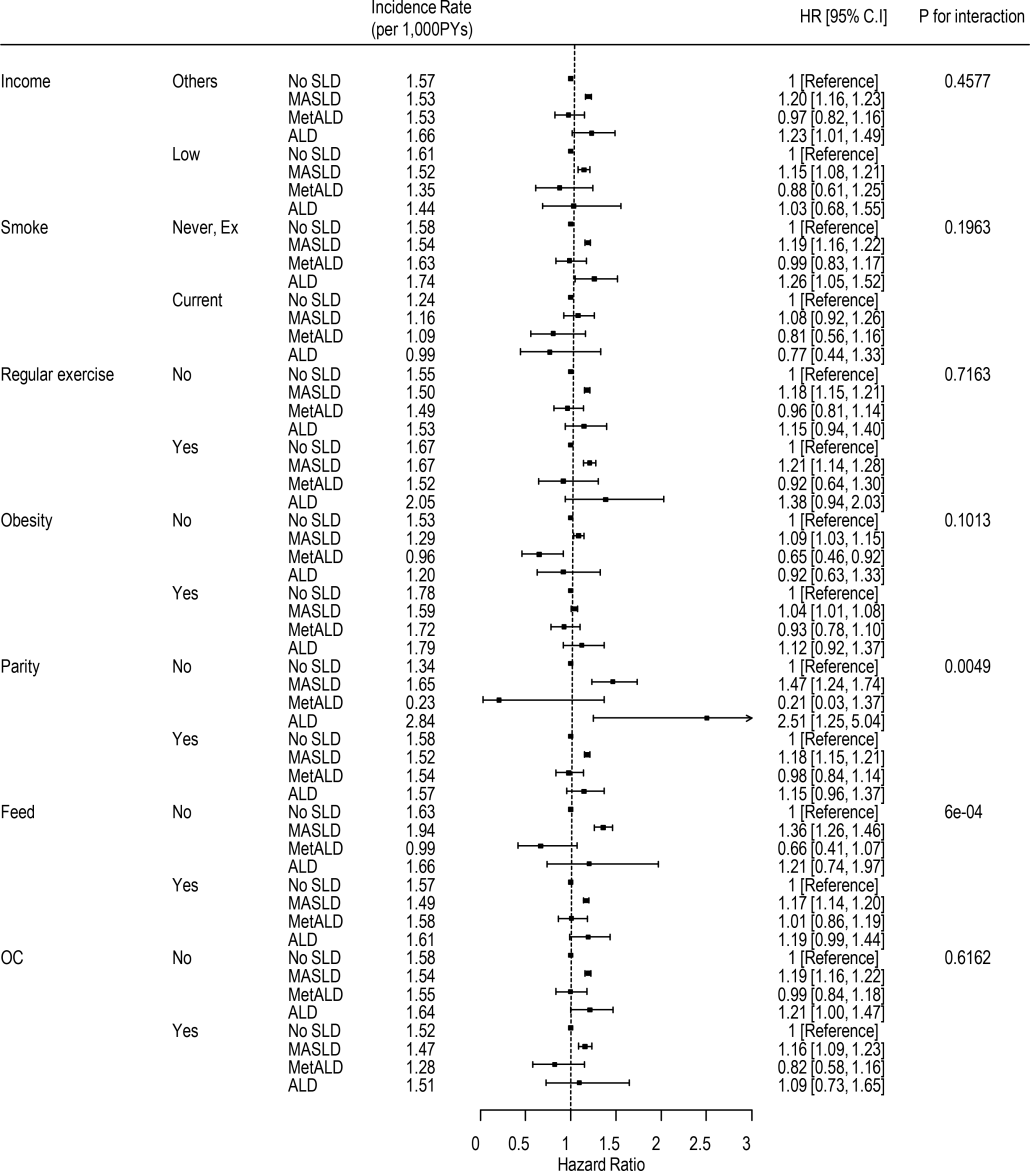
Subgroup analysis of the risk of thyroid cancer according to steatotic liver disease subtype. HR, hazard ratio; CI, confidence interval; SLD, steatotic liver disease; MASLD, metabolic dysfunction-associated steatotic liver disease; MetALD, metabolic alcohol-associated liver disease; ALD, alcohol-related liver disease.

The risk of thyroid cancer stratified by income, smoking status, obesity, parity, breastfeeding, and oral contraceptive use is shown in **Figure 2**. Across all subsets, MASLD was associated with a higher risk of thyroid cancer. However, MetALD was not significantly associated with the risk of thyroid cancer. The risk of thyroid cancer in the ALD group did not show a consistent trend in total participants. When premenopausal and postmenopausal women were analyzed separately, the ALD group had a higher risk of thyroid cancer than the no SLD group of postmenopausal women (**Supplementary Figure 2**). In premenopausal women, the ALD group was not associated with the risk of thyroid cancer in most subsets (**Supplementary Figure 3**).

## Discussion

This nationwide cohort study evaluated the association between SLD subtype and the risk of incident thyroid cancer in women aged over 40 years. During a mean follow-up of 12.27 years, the cumulative incidence of thyroid cancer in women with SLD was higher than that in those without SLD. Compared with the no SLD group, the MASLD group was associated with a greater risk of thyroid cancer, but the MetALD group was not, regardless of menopausal status. A different association was observed in the ALD group according to menopausal status. Postmenopausal women in the ALD group had a higher risk of thyroid cancer, while premenopausal women in the ALD group did not.

The incidence of MASLD is approximately 30% in the general population, and it is an emerging cause of liver-related morbidity worldwide. Risk factors for MASLD primarily include obesity, insulin resistance, and type 2 diabetes mellitus (25). These factors are also known as risk factors for cancers through mechanisms involving hormonal imbalance, chronic inflammation, and altered cell growth (26). Thus, MASLD might potentially be linked to an increased risk of cancer. For instance, Jeong et al. have reported that individuals with persistent MASLD have a higher risk of hepatocellular carcinoma (adjusted HR: 2.94, 95% CI: 2.68-3.21) than those with persistent non-MASLD. Similarly, Li et al. have reported that the MASLD group has an increased risk of colorectal cancer (HR: 1.54, 95% CI: 1.12-2.13) compared to those in the non-SLD group who are metabolically normal (27, 28). NAFLD, the former nomenclature for MASLD, has been associated with a higher risk of thyroid cancer. Kwon et al. reported that individuals with an FLI of 30 to 60 (HR: 1.39, 95% CI: 1.27-1.52) or an FLI ≥ 60 (HR: 1.75, 95% CI: 1.59-1.93) had a higher risk of thyroid cancer compared with those with an FLI < 30 (14). Recently, the term MASLD has been introduced to emphasize metabolic factors, and evidence regarding the relationship between MASLD and thyroid cancer remains limited (15). Therefore, we conducted this study to investigate their potential association.

MASLD is defined as SLD with at least one of the five cardiometabolic risk factors without excessive alcohol consumption (≥ 20 g/day) or concomitant liver disease. Additionally, two new categories, MetALD and ALD with cardiometabolic risk factors, were introduced to describe individuals with MASLD who consume greater amounts of alcohol (20–50 g/day and ≥ 50 g/day, respectively). In previous studies, obesity and NAFLD have been associated with a higher risk of thyroid cancer, whereas alcohol consumption has been inversely associated with thyroid cancer (14, 18). Thus, we hypothesized that the risk of thyroid cancer may vary by SLD subtype, as the new classification better reflects metabolic dysfunction and patterns of alcohol consumption. Our findings suggest that the risk of thyroid cancer was higher in the MASLD group, whereas no significant association was observed in the MetALD and ALD groups.

While metabolic factors such as obesity and insulin resistance have been associated with an increased risk of thyroid cancer, many large cohort studies have reported an inverse relationship between alcohol consumption and thyroid cancer risk, particularly in women (16–18, 29). Several potential mechanisms have been proposed to explain this inverse association. Alcohol consumption has been linked to decreased thyroid-stimulating hormone levels, which might reduce thyroid cell growth and lower the risk of thyroid carcinogenesis (30, 31). Additionally, alcoholic beverages such as red wine and beer contain polyphenols known to possess anticarcinogenic properties (32). For instance, a prospective case-control study has observed an inverse relationship between polyphenols and papillary thyroid cancer risk (33). In this context, the increased risk observed in MASLD may largely reflect the effect of metabolic dysfunction, such as insulin resistance, whereas the lack of a significant association in MetALD and ALD may be attributable to the counterbalancing effect of alcohol consumption. However, the mechanisms underlying these associations remain unclear.

In this study, we focused on female participants. The strength of the association between obesity and thyroid cancer risk appears to differ by sex (10, 34). In the Asian Cohort Consortium study, higher BMI was associated with an increased risk of thyroid cancer in both men and women, with a non-linear association seen in women whereas a linear association was seen in men (10). In addition, the incidence of thyroid cancer is approximately three to four times higher in women than in men, with age-specific incidence increasing steadily in men but peaking during the reproductive years in women (2, 35). These observations highlight the need to consider sex-specific biological mechanisms, particularly hormonal and reproductive influences, when interpreting thyroid cancer risk. As this study included only female participants, further research including men is warranted to confirm the generalizability of our findings.

Higher estrogen exposure has been suggested as a potential risk factor for thyroid cancer. Previous studies have reported that longer reproductive duration and later age at menopause are associated with an increased risk of thyroid cancer (36, 37). Based on this evidence, we stratified participants by menopausal status. In this study, inconsistencies in thyroid cancer risk among women with ALD were observed depending on menopausal status. ALD was not associated with thyroid cancer risk in premenopausal women (HR 0.98, 95% CI 0.73–1.30), whereas a modest association was observed in postmenopausal women (HR 1.31, 95% CI 1.05–1.64). However, the number of events within subgroups was limited, and the relatively wide confidence interval indicates limited precision of the estimate. Therefore, these findings should be interpreted with caution and not overinterpreted as evidence of a distinct biological mechanism. Further studies incorporating detailed reproductive history and sex hormone data are needed to clarify these associations.

Additionally, detection bias may have partially influenced our findings. The incidence of thyroid cancer can be higher due to increased healthcare utilization and surveillance intensity. Individuals with MASLD might undergo more health check-ups or imaging studies, which could lead to a greater incidental detection of thyroid cancer (38, 39). However, this alone may not fully explain the results, as patients with MetALD and ALD may also undergo frequent medical evaluations. Nonetheless, no significant association with thyroid cancer was observed in these groups. This suggests that differences in surveillance intensity alone may not fully explain our findings. Other factors could also contribute to the link between MASLD and thyroid cancer.

Several limitations should be considered when interpreting results of this study. First, SLD was defined using the FLI, which may be less accurate than ultrasonography or liver biopsy in assessing hepatic steatosis and may lead to misclassification. However, FLI is a cost-effective, non-invasive biomarker for predicting hepatic steatosis, and it has been validated in various populations, including the Korean population (40, 41). In addition, the FLI was calculated based on metabolic components such as BMI, waist circumference, triglycerides, and GGT. Therefore, the observed association between MASLD and thyroid cancer risk may be partially influenced by metabolic confounding. Second, information on smoking status and alcohol consumption was based on self-reported questionnaires, which might have led to underestimation or overestimation. ALD was defined based on self-reported excessive alcohol intake or alcohol-related diagnostic codes. However, discrepancies between diagnostic codes and self-reported alcohol consumption were observed. In this study, 49.8% of individuals classified as ALD reported no alcohol consumption, and 16.2% reported mild consumption. This inconsistency may reflect underreporting or past alcohol exposure. Third, age at menarche, age at menopause, duration of breastfeeding, duration of HRT, and duration of oral contraceptive use were collected through self-reported questionnaires, which might be subject to a recall bias, particularly among older participants. Fourth, information on pathological type and stage of thyroid cancer was not available. Furthermore, data on actual thyroid function were not available. This lack of detailed thyroid-related variables may limit the biological interpretation and clinical relevance of our findings. However, given that papillary thyroid cancer accounts for approximately 97% of cases in Korea, other cancer subtypes are likely to be underrepresented in this study (42). Fifth, as this study was conducted in a Korean population with an iodine-rich diet, caution is required when generalizing these findings to other populations or ethnic groups (43). Finally, although the association between MASLD and thyroid cancer was statistically significant, the effect size was modest, suggesting limited clinical relevance at the individual level. However, given the high prevalence of MASLD, these findings may still be meaningful at the population level. Despite these limitations, this is the first large-scale cohort study to assess the relationship between the risk of thyroid cancer and newly categorized SLD subtypes in women stratified by menopausal status.

## Conclusions

MASLD was associated with a higher risk of thyroid cancer in women over 40 years old, whereas MetALD and ALD were not. However, further studies are needed to better understand these associations.

## Data Availability

The datasets generated and/or analyzed during the current study are not publicly available due to patient privacy and institutional restrictions but are available from the corresponding author upon reasonable request.
