# Supplementary material for: Association between steatotic liver disease subtype and thyroid cancer in women: A nationwide population-based cohort study
Source: Front Endocrinol (Lausanne). 2026 May 21;17:1801839. doi: 10.3389/fendo.2026.1801839 (PMC13233242; doi:10.3389/fendo.2026.1801839)

Supplementary Table 1. Baseline characteristics according to the presence of steatotic liver disease

| Characteristics | Total  (n=2,089,609) | No SLD  (n=1,597,364) | SLD  (n=492,245) | P value |
| --- | --- | --- | --- | --- |
| Age, years | 54.8 ± 10.8 | 53.5 ± 10.6 | 59.0 ± 10.3 | < 0.001 |
| BMI, kg/m^2^ | 23.78 ± 3.15 | 22.73 ± 2.38 | 27.2 ± 2.9 | < 0.001 |
| Waist circumference, cm | 78.0 ± 8.4 | 75.2 ± 6.5 | 87.3 ± 7.0 | < 0.001 |
| Smoking status |  |  |  | < 0.001 |
| Never | 2,001,499 (95.78) | 1,533,826 (96.02) | 467,673 (95.01) |  |
| Former | 26,449 (1.27) | 19,911 (1.25) | 6,538 (1.33) |  |
| Current | 61,661 (2.95) | 43,627 (2.73) | 18,034 (3.66) |  |
| Alcohol consumption |  |  |  | < 0.001 |
| None | 1,692,185 (80.98) | 1,282,538 (80.29) | 409,647 (83.22) |  |
| Mild | 362,939 (17.37) | 291,521 (18.25) | 71,418 (14.51) |  |
| Moderate | 29,801 (1.43) | 20,414 (1.28) | 9,387 (1.91) |  |
| Heavy | 4,684 (0.22) | 2,891 (0.18) | 1,793 (0.36) |  |
| Regular exercise | 372,125 (17.81) | 293,440 (18.37) | 78,685 (15.98) | < 0.001 |
| Low income | 392,919 (18.80) | 304,605 (19.07) | 88,314 (17.94) | < 0.001 |
| Obesity | 668,290 (31.98) | 281,264 (17.61) | 387,026 (78.62) | < 0.001 |
| Diabetes mellitus | 186,244 (8.91) | 92,780 (5.81) | 93,464 (18.99) | < 0.001 |
| Hypertension | 677,549 (32.42) | 405,289 (25.37) | 272,260 (55.31) | < 0.001 |
| Dyslipidemia | 490,815 (23.49) | 295,850 (18.52) | 194,965 (39.61) | < 0.001 |
| SBP, mmHg | 122.2 ± 16.0 | 120.1 ± 15.4 | 129.2 ± 16.0 | < 0.001 |
| DBP, mmHg | 75.4 ± 10.3 | 74.2 ± 10.0 | 79.2 ± 10.2 | < 0.001 |
| Fasting glucose, mg/dL | 97.2 ± 22.1 | 94.7 ± 18.7 | 105.3 ± 29.2 | < 0.001 |
| Total cholesterol, mg/dL | 201.6 ± 37.7 | 197.8 ± 36.0 | 213.8 ± 40.5 | < 0.001 |
| Triglyceride, mg/dL | 104.14 (104.07-104.22) | 89.99 (89.92-90.05) | 167.30 (167.08-167.52) | < 0.001 |
| HDL cholesterol, mg/dL | 58.6 ± 32.1 | 59.7 ± 29.8 | 54.8 ± 38.2 | < 0.001 |
| LDL cholesterol, mg/dL | 120.5 ± 37.8 | 119.4 ± 36.3 | 124.3 ± 42.0 | < 0.001 |
| eGFR, mL/min/1.73m2 | 84.88 ± 28.76 | 85.74 ± 28.64 | 82.1 ± 29.0 | < 0.001 |
| AST, IU/L | 22.32 (22.31-22.33) | 21.45 (21.44-21.46) | 25.41 (25.39-25.44) | < 0.001 |
| ALT, IU/L | 18.55 (18.54-18.57) | 17.00 (16.99-17.01) | 24.66 (24.62-24.70) | < 0.001 |
| r-GTP, IU/L | 19.12 (19.11-19.14) | 16.57 (16.56-16.59) | 30.41 (30.36-30.47) | < 0.001 |
| Age at menarche, years | 15.9 ± 1.9 | 15.8 ± 1.9 | 16.2 ± 1.9 | < 0.001 |
| Menopause | 1,220,141 (58.39) | 845,856 (52.95) | 374,285 (76.04) | < 0.001 |
| Parity |  |  |  | < 0.001 |
| No | 51,866 (2.48) | 43,099 (2.70) | 8,767 (1.78) |  |
| 1 | 187,123 (8.95) | 154,376 (9.66) | 32,747 (6.65) |  |
| ≥ 2 | 1,850,620 (88.56) | 1,399,889 (87.64) | 450,731 (91.57) |  |
| Breastfeeding, months |  |  |  | < 0.001 |
| 0 | 232,798 (11.14) | 193,473 (12.11) | 39,325 (7.99) |  |
| < 6 | 289,595 (13.86) | 251,091 (15.72) | 38,504 (7.82) |  |
| 6 – 12 | 436,010 (20.87) | 353,369 (22.12) | 82,641 (16.79) |  |
| ≥ 12 | 1,131,206 (54.13) | 799,431 (50.05) | 331,775 (67.40) |  |
| Oral contraceptive use, months |  |  |  | < 0.001 |
| 0 | 1,789,179 (85.62) | 1,375,807 (86.13) | 413,372 (83.98) |  |
| < 12 | 195,858 (9.37) | 148,443 (9.29) | 47,415 (9.63) |  |
| ≥ 12 | 104,572 (5.00) | 73,114 (4.58) | 31,458 (6.39) |  |

Continuous variables are expressed as mean ± standard deviation or geometric mean (95% confidential interval). Categorical data are presented as frequencies and percentages.

BMI, body mass index; SBP, systolic blood pressure; DBP, diastolic blood pressure; HDL, high-density lipoprotein; LDL, low-density lipoprotein; eGFR, estimated glomerular filtration rate; AST, aspartate aminotransferase; ALT, alanine aminotransferase; r-GTP, gamma-glutamyl transferase; SLD, steatotic liver disease.

Supplementary Table 2. Baseline characteristics according to steatotic liver disease subtype stratified by menopausal status

|  | Premenopausal women | | | | | | Postmenopausal women | | | | | |
| --- | --- | --- | --- | --- | --- | --- | --- | --- | --- | --- | --- | --- |
| Characteristics | Total  (n=869,468) | No SLD  (n=751,508) | MASLD  (n=111,247) | MetALD  (n=4,671) | ALD  (n=2,042) | P value | Total  (n=1,220,141) | No SLD  (n=845,856) | MASLD  (n=365,627) | MetALD  (n=4,346) | ALD  (n=4,312) | P value |
| Age, years | 45.1 ± 4.2 | 44.9 ± 4.1 | 46.2 ± 4.9 | 45.5 ± 4.0 | 46.0 ± 4.3 | <.0001 | 61.7 ± 8.5 | 61.1 ± 8.6 | 63.1 ± 8.0 | 57.7 ± 7.1 | 61.0 ± 8.0 | <.0001 |
| BMI, kg/m^2^ | 23.22 ± 3.06 | 22.51 ± 2.37 | 27.76 ± 3.13 | 26.75 ± 3.32 | 26.57 ± 3.54 | < 0.001 | 24.18 ± 3.15 | 22.92 ± 2.36 | 27.04 ± 2.83 | 26.51 ± 3.11 | 26.63 ± 3.07 | < 0.001 |
| Waist circumference, cm | 75.2 ± 7.7 | 73.5 ± 6.2 | 86.3 ± 7.2 | 85.3 ± 7.6 | 84.9 ± 7.9 | < 0.001 | 80.1 ± 8.3 | 76.7 ± 6.4 | 87.7 ± 6.8 | 86.4 ± 7.4 | 86.9 ± 7.4 | < 0.001 |
| Smoking status |  |  |  |  |  | < 0.001 |  |  |  |  |  | < 0.001 |
| Never | 826,622 (95.07) | 717,171 (95.43) | 104,975 (94.36) | 3,084 (66.02) | 1,392 (68.17) |  | 1,174,877 (96.29) | 816,655 (96.55) | 351,309 (96.08) | 3,202 (73.68) | 3,711 (86.06) |  |
| Former | 13,751 (1.58) | 11,620 (1.55) | 1,771 (1.59) | 273 (5.84) | 87 (4.26) |  | 12,698 (1.04) | 8,291 (0.98) | 4,094 (1.12) | 211 (4.86) | 102 (2.37) |  |
| Current | 29,095 (3.35) | 22,717 (3.02) | 4,501 (4.05) | 1,314 (28.13) | 563 (27.57) |  | 32,566 (2.67) | 20,910 (2.47) | 10,224 (2.8) | 933 (21.47) | 499 (11.57) |  |
| Alcohol consumption status |  |  |  |  |  | < 0.001 |  |  |  |  |  | < 0.001 |
| None | 622,922 (71.64) | 540,704 (71.95) | 81,655 (73.40) | 0 (0.00) | 563 (27.57) |  | 1,069,263 (87.63) | 741,834 (87.70) | 324,830 (88.84) | 0 (0.00) | 2,599 (60.27) |  |
| Mild | 224,919 (25.87) | 194,959 (25.94) | 29,592 (26.60) | 0 (0.00) | 368 (18.02) |  | 138,020 (11.31) | 96,562 (11.42) | 40,797 (11.16) | 0 (0.00) | 661 (15.33) |  |
| Moderate | 18,818 (2.16) | 13,977 (1.86) | 0 (0.00) | 4,671 (100.00) | 170 (8.33) |  | 10,983 (0.90) | 6,437 (0.76) | 0 (0.00) | 4,346 (100.00) | 200 (4.64) |  |
| Heavy | 2,809 (0.32) | 1,868 (0.25) | 0 (0.00) | 0 (0.00) | 941 (46.08) |  | 1,875 (0.15) | 1,023 (0.12) | 0 (0.00) | 0 (0.00) | 852 (19.76) |  |
| Regular exercise | 149,744 (17.22) | 131,728 (17.53) | 16,855 (15.15) | 822 (17.60) | 339 (16.60) | < 0.001 | 222,381 (18.23) | 161,712 (19.12) | 59,128 (16.17) | 843 (19.4) | 698 (16.19) | < 0.001 |
| Low income | 175,627 (20.2) | 151,482 (20.16) | 22,665 (20.37) | 993 (21.26) | 487 (23.85) | < 0.001 | 217,292 (17.81) | 153,123 (18.10) | 62,477 (17.09) | 881 (20.27) | 811 (18.81) | < 0.001 |
| Obesity | 213,626 (24.57) | 116,779 (15.54) | 92,188 (82.87) | 3,299 (70.63) | 1360 (66.60) | < 0.001 | 454,664 (37.26) | 164,485 (19.45) | 284,084 (77.70) | 3,002 (69.08) | 3,093 (71.73) | < 0.001 |
| Diabetes mellitus | 30,400 (3.50) | 16,605 (2.21) | 13,051 (11.73) | 460 (9.85) | 284 (13.91) | < 0.001 | 155,844 (12.77) | 76,175 (9.01) | 77,785 (21.27) | 740 (17.03) | 1,144 (26.53) | < 0.001 |
| Hypertension | 120,987 (13.92) | 81,905 (10.90) | 36,499 (32.81) | 1,707 (36.54) | 876 (42.9) | < 0.001 | 556,562 (45.61) | 323,384 (38.23) | 227,889 (62.33) | 2,467 (56.76) | 2,822 (65.45) | < 0.001 |
| Dyslipidemia | 93,299 (10.73) | 64,104 (8.53) | 27,555 (24.77) | 1,023 (21.9) | 617 (30.22) | < 0.001 | 397,516 (32.58) | 231,746 (27.4) | 161,738 (44.24) | 1,768 (40.68) | 2,264 (52.50) | < 0.001 |
| SBP, mmHg | 117.3 ± 14.4 | 116.0 ± 13.7 | 125.5 ± 15.3 | 127.1 ± 15.8 | 126.0 ± 15.5 | < 0.001 | 125.7 ± 16.2 | 123.7 ± 15.9 | 130.3 ± 16.0 | 130.7 ± 16.4 | 129.8 ± 16.1 | < 0.001 |
| DBP, mmHg | 73.2 ± 10.0 | 72.3 ± 9.6 | 78.5 ± 10.4 | 80.2 ± 10.9 | 79.5 ± 10.6 | < 0.001 | 77.0 ± 10.2 | 75.9 ± 10.0 | 79.4 ± 10.1 | 80.8 ± 10.5 | 79.7 ± 10.3 | < 0.001 |
| Fasting glucose, mg/dL | 93.7 ± 17.9 | 92.2 ± 15.3 | 102.7 ± 27.9 | 103.3 ± 26.2 | 104.9 ± 28.6 | < 0.001 | 99.7 ± 24.3 | 96.9 ± 21.0 | 106.0 ± 29.5 | 108.0 ± 29.7 | 109.1 ± 30.7 | < 0.001 |
| Total cholesterol, mg/dL | 192.1 ± 34.0 | 189.6 ± 32.7 | 207.9 ± 37.8 | 206.2 ± 36.8 | 205.2 ± 40.2 | < 0.001 | 208.3 ± 38.7 | 205.1 ± 37.2 | 215.7 ± 41.0 | 220.3 ± 41.2 | 212.6 ± 43.2 | < 0.001 |
| Triglyceride, mg/dL | 88.68 (88.58-88.77) | 80.77 (80.69-80.86) | 160.28 (159.82-160.74) | 166.35 (163.97-168.78) | 172.42 (168.63-176.29) | < 0.001 | 116.78 (116.67-116.89) | 99.05 (98.96-99.15) | 169.47 (169.21-169.72) | 167.5 (165.03-170.01) | 167.79(165.36-170.26) | < 0.001 |
| HDL cholesterol, mg/dL | 60.0 ± 30.1 | 60.6 ± 26.9 | 56.0 ± 45.6 | 62.1 ± 39.5 | 60.2 ± 36.4 | < 0.001 | 57.6 ± 33.3 | 59.0 ± 32.1 | 54.3 ± 35.7 | 60.9 ± 31.7 | 57.0 ± 35.6 | < 0.001 |
| LDL cholesterol, mg/dL | 113.2 ± 35.6 | 112.2 ± 34.4 | 119.9 ± 42.2 | 108.3 ± 37.5 | 108.3 ± 47.1 | < 0.001 | 125.8 ± 38.3 | 125.7 ± 36.7 | 125.9 ± 41.8 | 122.5 ± 40.9 | 119.5 ± 42.4 | < 0.001 |
| eGFR, mL/min/1.73m2 | 89.03 ± 29.04 | 89.18 ± 28.9 | 88.00 ± 30.03 | 89.92 ± 25.66 | 89.27 ± 30.67 | < 0.001 | 81.92 ± 28.19 | 82.69 ± 28.06 | 80.13 ± 28.35 | 85.31 ± 32.81 | 81.05 ± 25.98 | < 0.001 |
| AST, IU/L | 20.40 (20.39-20.42) | 19.88 (19.86-19.89) | 23.96 (23.91-24.02) | 25.58 (25.25-25.91) | 28.68 (28.02-29.35) | < 0.001 | 23.80 (23.78-23.81) | 22.95 (22.93-22.96) | 25.77 (25.74-25.80) | 28.22 (27.85-28.59) | 30.03 (29.59-30.48) | < 0.001 |
| ALT, IU/L | 16.62 (16.61-16.64) | 15.63 (15.61-15.64) | 24.62 (24.54-24.70) | 24.14 (23.78-24.51) | 27.53 (26.81-28.26) | < 0.001 | 20.06 (20.05-20.08) | 18.31 (18.30-18.33) | 24.61 (24.57-24.65) | 25.67 (25.28-26.07) | 28.08 (27.61-28.56) | < 0.001 |
| r-GTP, IU/L | 17.21 (17.19-17.23) | 15.66 (15.65-15.68) | 30.48 (30.37-30.59) | 47.23 (46.25-48.22) | 55.07 (53.04-57.18) | < 0.001 | 20.62 (20.60-20.64) | 17.43 (17.41-17.45) | 29.80 (29.74-29.86) | 49.11 (48.03-50.22) | 46.52 (45.38-47.70) | < 0.001 |
| Age at menarche, years | 15.1 ± 1.7 | 15.1 ± 1.7 | 15.2 ± 1.8 | 15.4 ± 1.8 | 15.5 ± 1.8 | < 0.001 | 16.5 ± 1.8 | 16.4 ± 1.9 | 16.6 ± 1.8 | 16.5 ± 1.9 | 16.6 ± 1.9 | < 0.001 |
| Age at Menopause, years | N/A | N/A | N/A | N/A | N/A |  | 50.0 ± 4.0 | 49.9 ± 3.9 | 50.1 ± 4.2 | 49.9 ± 4.2 | 50.0 ± 4.2 | < 0.001 |
| Parity |  |  |  |  |  | < 0.001 |  |  |  |  |  | < 0.001 |
| No | 31,638 (3.64) | 27,826 (3.70) | 3,472 (3.12) | 219 (4.69) | 121 (5.93) |  | 20,228 (1.66) | 15,273 (1.81) | 4,712 (1.29) | 132 (3.04) | 111 (2.57) |  |
| 1 | 115,106 (13.24) | 99,613 (13.26) | 14,348 (12.9) | 795 (17.02) | 350 (17.14) |  | 72,017 (5.90) | 54,763 (6.47) | 16,490 (4.51) | 462 (10.63) | 302 (7.00) |  |
| ≥ 2 | 722,724 (83.12) | 624,069 (83.04) | 93,427 (83.98) | 3,657 (78.29) | 1,571 (76.93) |  | 1,127,896 (92.44) | 775,820 (91.72) | 344,425 (94.2) | 3,752 (86.33) | 3,899 (90.42) |  |
| Breastfeeding, months |  |  |  |  |  | < 0.001 |  |  |  |  |  | < 0.001 |
| 0 | 155,423 (17.88) | 135,213 (17.99) | 18,806 (16.90) | 946 (20.25) | 458 (22.43) |  | 77,375 (6.34) | 58,260 (6.89) | 18,337 (5.02) | 448 (10.31) | 330 (7.65) |  |
| < 6 | 213,764 (24.59) | 191,411 (25.47) | 21,125 (18.99) | 887 (18.99) | 341 (16.70) |  | 75,831 (6.21) | 59,680 (7.06) | 15,688 (4.29) | 273 (6.28) | 190 (4.41) |  |
| 6 – 12 | 229,224 (26.36) | 200,501 (26.68) | 27,263 (24.51) | 1,018 (21.79) | 442 (21.65) |  | 206,786 (16.95) | 152,868 (18.07) | 52,647 (14.40) | 682 (15.69) | 589 (13.66) |  |
| ≥ 12 | 271,057 (31.18) | 224,383 (29.86) | 44,053 (39.60) | 1,820 (38.96) | 801 (39.23) |  | 860,149 (70.50) | 575,048 (67.98) | 278,955 (76.29) | 2,943 (67.72) | 3,203 (74.28) |  |
| Oral contraceptive use, months |  |  |  |  |  | < 0.001 |  |  |  |  |  | < 0.001 |
| 0 | 757,179 (87.09) | 656,783 (87.40) | 95,321 (85.68) | 3,510 (75.14) | 1,565 (76.64) |  | 1,032,000 (84.58) | 719,024 (85.01) | 306,022 (83.7) | 3,408 (78.42) | 3,546 (82.24) |  |
| < 12 | 82,682 (9.51) | 70,571 (9.39) | 11,151 (10.02) | 681 (14.58) | 279 (13.66) |  | 113,176 (9.28) | 77,872 (9.21) | 34,388 (9.41) | 492 (11.32) | 424 (9.83) |  |
| ≥ 12 | 29,607 (3.41) | 24,154 (3.21) | 4,775 (4.29) | 480 (10.28) | 198 (9.70) |  | 74,965 (6.14) | 48,960 (5.79) | 25,217 (6.90) | 446 (10.26) | 342 (7.93) |  |
| Hormone replacement theraphy, years |  |  |  |  |  |  |  |  |  |  |  | < 0.001 |
| 0 | N/A | N/A | N/A | N/A | N/A |  | 1,026,599 (84.14) | 698,183 (82.54) | 321,210 (87.85) | 3,553 (81.75) | 3,653 (84.72) |  |
| < 2 | N/A | N/A | N/A | N/A | N/A |  | 112,712 (9.24) | 84,229 (9.96) | 27,623 (7.55) | 471 (10.84) | 389 (9.02) |  |
| 2 - 5 | N/A | N/A | N/A | N/A | N/A |  | 45,957 (3.77) | 36,052 (4.26) | 9,567 (2.62) | 184 (4.23) | 154 (3.57) |  |
| ≥ 5 | N/A | N/A | N/A | N/A | N/A |  | 34,873 (2.86) | 27,392 (3.24) | 7,227 (1.98) | 138 (3.18) | 116 (2.69) |  |

Continuous variables are expressed as mean ± standard deviation or geometric mean (95% confidential interval). Categorical data are presented as frequencies and percentages.

BMI, body mass index; SBP, systolic blood pressure; DBP, diastolic blood pressure; HDL, high-density lipoprotein; LDL, low-density lipoprotein; eGFR, estimated glomerular filtration rate; AST, aspartate aminotransferase; ALT, alanine aminotransferase; r-GTP, gamma-glutamyl transferase; SLD, steatotic liver disease; MASLD, metabolic dysfunction-associated steatotic liver disease; MetALD, metabolic alcohol-associated liver disease; ALD, alcohol-related liver disease.


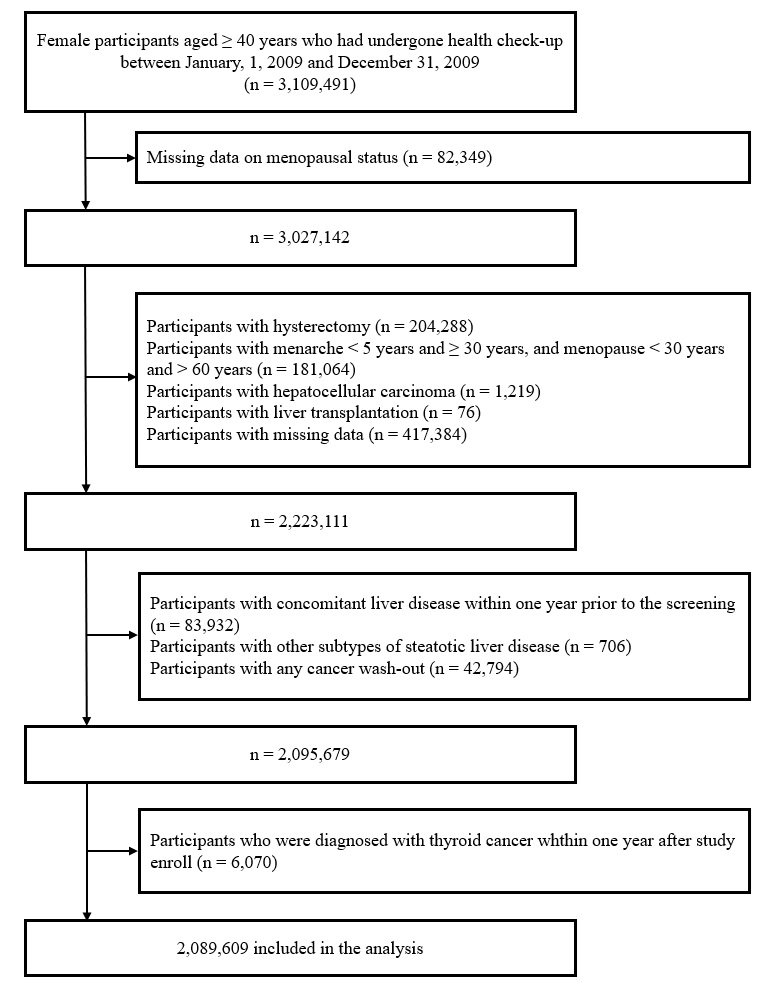
Supplementary figure 1. Flow chart of the patient selection process.

Supplementary Figure 2. Subgroup analysis of the risk of thyroid cancer according to steatotic liver disease subtype in premenopausal women (HR, hazard ratio; CI, confidence interval; SLD, steatotic liver disease; MASLD, metabolic dysfunction-associated steatotic liver disease; MetALD, metabolic alcohol-associated liver disease; ALD, alcohol-related liver disease).


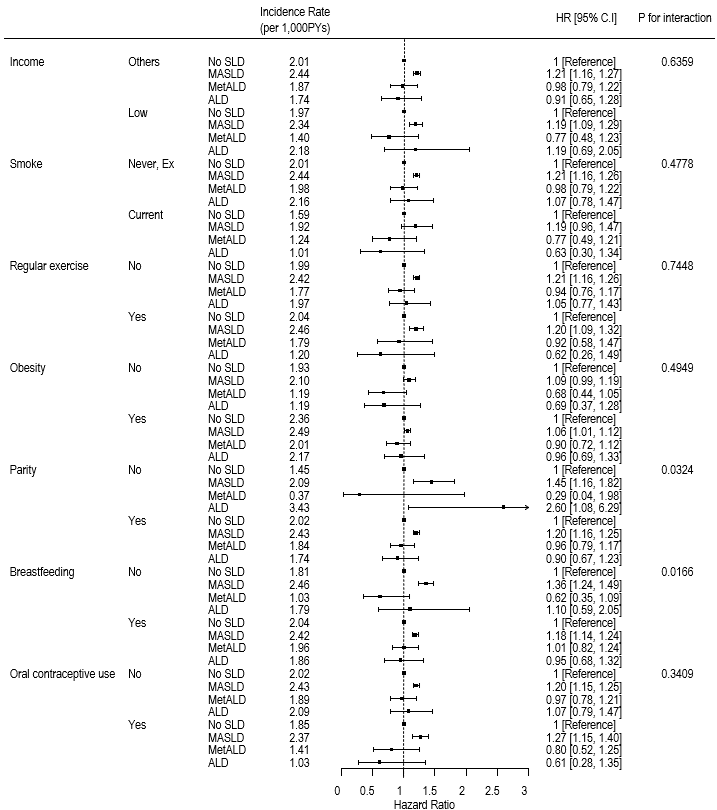


Supplementary Figure 3. Subgroup analysis of the risk of thyroid cancer according to steatotic liver disease subtype in postmenopausal women (HR, hazard ratio; CI, confidence interval; SLD, steatotic liver disease; MASLD, metabolic dysfunction-associated steatotic liver disease; MetALD, metabolic alcohol-associated liver disease; ALD, alcohol-related liver disease).


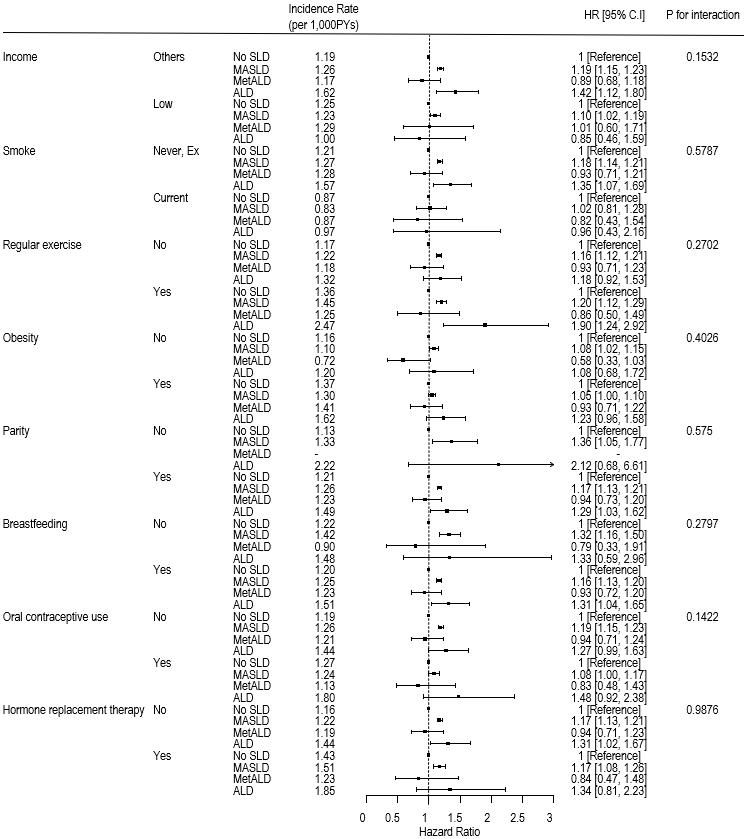

Supplement: Supplementary file 1 [file DataSheet1.docx]
